# Phytochemical Screening of Quaking Aspen (*Populus tremuloides*) Extracts by UPLC-QTOF-MS and Evaluation of their Antimicrobial Activity

**DOI:** 10.3390/molecules23071739

**Published:** 2018-07-16

**Authors:** Annabelle St-Pierre, Dorian Blondeau, André Lajeunesse, Julien Bley, Nathalie Bourdeau, Isabel Desgagné-Penix

**Affiliations:** 1 Department of Chemistry, Biochemistry and Physics, Université du Québec à Trois-Rivières, Trois-Rivières, QC G9A 5H7, Canada; Annabelle.St-Pierre@uqtr.ca (A.S.-P.); Dorian.Blondeau@uqtr.ca (D.B.); Andre.Lajeunesse@uqtr.ca (A.L.); 2 Innofibre, Trois-Rivières, QC G9A 5H7, Canada; julien.bley@cegeptr.qc.ca (J.B.); nathalie.bourdeau@cegeptr.qc.ca (N.B.); 3 Plant Biology Research Group, Trois-Rivières, QC G9A 5H7, Canada

**Keywords:** quaking aspen, *Populus tremuloides*, antimicrobial activity, UPLC-QTOF-MS, phenolic compounds, flavonoids

## Abstract

The continual emergence of pathogen resistance is a recurring challenge and pushes for the development of antimicrobial compounds. Here, we investigated compounds from quaking aspen trees (*Populus tremuloides*) as potential antimicrobial agents. Several extractions using different solvents were realized, and corresponding antimicrobial activity was tested against eight microorganisms. Results revealed that polar extraction solvents including water, ethanol and methanol gave the best extraction yields (>15.07%). Minimal inhibition concentration (MIC) and minimal bactericidal/fungicidal concentration (MBC/MFC) demonstrated that water extracts had the best antimicrobial activity by a weak to moderate inhibition of growth of all eight tested microorganisms in addition to having a bactericidal effect on three of them. The quaking aspen methanol extract also displayed antimicrobial activity but to a lower level than the water extract. Ultra-performance liquid chromatography quadrupole time-of flight mass spectrometry (UPLC-QTOF-MS) analysis led to the identification of 92 compounds, mainly polyphenols in both extracts, with 22 molecules previously known for their antimicrobial properties. According to the relative abundance, 4-hydroxybenzaldehyde (5.44% in methanol extract) and kaempferol (5.03% in water extract) were the most abundant antimicrobial compounds. Among antimicrobial molecules identified, nine were from the flavonoid family. The results of our study demonstrate the interest of using quaking aspen as source of antimicrobial compounds.

## 1. Introduction

Continual emergence of pathogens resistant to antibiotics, antiseptics and disinfectants is a real challenge and it motivates innovation in the field of antimicrobial agent development [1,2]. This research field is in perpetual expansion in order to discover new molecules with antimicrobial activity. Although synthetic antimicrobial agent development has largely contributed to the field, the current environmental concerns coupled with the need to deal with our natural resource residues has prompted a renewed interest in natural antimicrobial molecules. Thus, it is relevant to look at plant specialized (*aka* secondary) metabolites known for their biological and therapeutic activities. Indeed, several current studies are using this alternative approach to investigate molecules produced by plants with the purpose of developing antimicrobial agents. In fact, it was estimated that there are around 25% of prescribed drugs on the market that come from plant metabolites [3,4].

Quaking aspen (*Populus tremuloides*) is a tree largely present in the boreal forests of North America and Eurasia and is poorly studied and exploited for its biological properties. This broadleaf tree exhibits the largest spatial distribution of all deciduous species in Canada. It stretches from Newfoundland and Labrador to British Columbia and Yukon [5]. Quaking aspen was formerly used in traditional medicine by Native American nations of Québec for its various therapeutic functions, either as a vermifuge, to treat venereal disease, as an anti-arthritis agent or as a cure for colds [5,6]. Since some of these applications suggest antimicrobial activities, quaking aspen has potential in the development of antimicrobial agents through the extraction of its specialized metabolites. Previous studies on the chemical composition of quaking aspen have revealed that this species is an important source of extractive compounds, i.e., molecules easily extractive with a solvent. It was reported that quaking aspen trees are rich in polyphenols, but also in sterols, steryls, triterpenes and alkaloids [7,8,9]. Polyphenols and alkaloids are families of compounds highly sought for their antioxidant, antitumor and antimicrobial activities [10,11,12,13]. These classes of compounds are recognized to be synthesized by plants to protect themselves against biotic stress. These molecules, according to their chemical structure, can act as toxins that alter biological processes of predators such as herbivores, fungi or bacteria [14,15]. Triterpenes are also known for their antimicrobial potential and are often involved in plant defense mechanisms against pathogens [16,17,18]. Currently, there are very few studies linking specialized (*aka* secondary) metabolites of quaking aspen to biological activities of interest. It would be advantageous to characterize specialized metabolites of this species and their respective antimicrobial efficiency.

The residual biomass of quaking aspen still remains sparsely exploited and valued while it is very abundant and easily accessible. Currently in Québec, this tree is mainly used by the forest industry for its transformation into pulp and paper, lumber and building materials [5,6]. During the industrial process of sawing wood, residues such as bark, knots and small branches are removed from the sapwood and discarded because of high content of undesirable compounds (lignins, polyphenols and resins) which contribute to low mechanical properties in the manufacture of lumber [19]. In this way, a significant volume of wood residues remains unexploited while they contain a large proportion of interesting metabolites. Since bark residues contain a lot of lignocellulosic compounds rich in organic matter, they are commonly burned and used as a bioenergy source. In some cases, bark ends up buried in the ground and is not valued [20,21]. Thus, it is necessary to focus on quaking aspen in the context of forest residue management and valorisation through the potential antimicrobial activities that its metabolites can offer.

Thus, the objective of this study was to evaluate the antimicrobial potential of quaking aspen through its active specialized metabolites extracted from bark residues. To achieve this goal, the general composition and extractive yield of different fraction sizes of bark residues were determined, and multiple solvents extractions for optimal yield of bioactive metabolites were performed. Next, the families of compounds present in the extracts were screened qualitatively using thin layer chromatography (TLC) analysis, and specific compounds were identified using ultra-performance liquid chromatography time of flight quadrupole mass spectrometry (UPLC-QTOF-MS) analysis. Antimicrobial activity was then evaluated in vitro by determining the minimal inhibition concentration (MIC) and the minimal bactericidal/fungicidal concentration (MBC/MFC) of extracts. These were evaluated on different bacterial and fungal strains including three Gram-negative bacteria, two Gram-positive bacteria and three fungi (two yeasts and one mold). The comparative analysis of the chemical composition of the extracts with their antimicrobial activity supports the use of quaking aspen as a potential antimicrobial source.

## 2. Results

### 2.1. Determination of Bark Composition and Extractive Yield per Fraction Size

**Figure 1:**
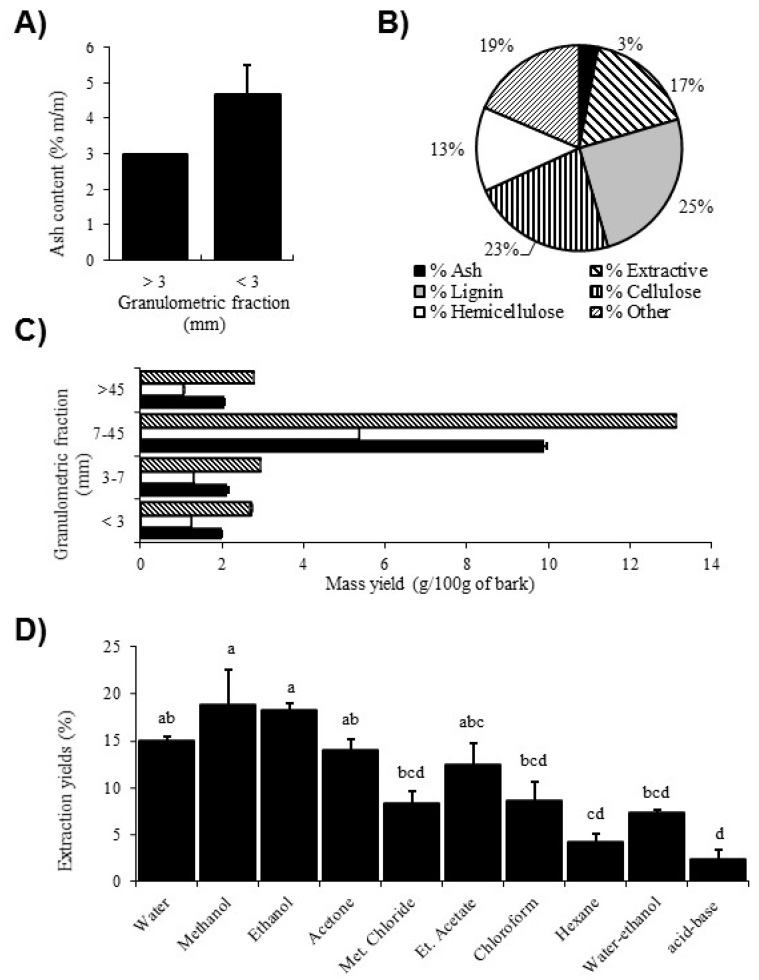
Evaluation of Quaking Aspen bark composition and yield according to different extraction parameters. (**A**) Ash content (% m/m) of bark according to the granulometric fraction <3 mm or >3 mm; (**B**) Quaking aspen bark relative composition of extractives, ash and lignocellulosic materials (% m/m); (**C**) Extraction yields of quaking aspen bark under different granulometric conditions and with different extraction solvents; (**D**) Mass yield (% of total dry mass) of quaking aspen bark according to different extraction solvents. In graph, columns identified with different letters (a–d) are significantly different using one-way analysis of variance (ANOVA) followed by pairwise mean comparison Tukey’s test where differences were detected (*n* = 3, *p* < 0.05).

First, the content of quaking aspen bark residues was evaluated. The measure of ash content provides an indication of the undesirable inorganic components present in wood. These components are not extractive material and their abundance can affect the extractive yield. As shown in Figure 1A, the ash content in the fraction size < 3 mm was higher than in the fraction greater than 3 mm, with an average proportion of 4.67% m/m and 2.97%, respectively.

An evaluation of the relative composition of the ash, lignin, cellulose, hemicellulose and extractive contents of bark was also carried out according to National renewable energy laboratory (NREL) protocols on each fraction greater than 3 mm. In Figure 1B, the results show that the bark residues had a significant amount of lignin (25% m/m) and cellulose (23% m/m), a non-negligible amount of extractive material (17% m/m) and, conversely, a low amount of ash (3% m/m).

To identify which fraction size contained the largest proportion of extractives to further optimize the yield, the bark of quaking aspen was sorted into different granulometric fractions (<3, 3–7, 7–45 and >45 mm) before being extracted with three solvents (water, ethanol and water-ethanol). The extractive mass yield (g/100 g) obtained for the different granulometric fractions is summarized in Figure 1C. The results show that the 7–45 mm fraction yielded a greater proportion of extractive materials compared to other fractions, and this result was observed for the three different extraction solvents. The fraction <3 mm generally showed the lowest yield compared to the 3–7 mm and >45 mm fractions.

### 2.2. Determination of Extractive Yield According to Molecule Polarity

The extraction was carried out with solvents of different polarities (water, methanol, ethanol, acetone, ethyl acetate, chloroform, methyl chloride, hexane, water-ethanol and acid-base extraction), in order to extract most of the specialized metabolites. The results in Figure 1D indicate that the solvents generating the highest yields were polar solvents with methanol being the highest, followed by ethanol and water. Methanol and ethanol extractions produced a significantly higher yield than the less polar solvents (chloroform, methylene chloride and hexane) and the multistage extraction method (water-ethanol and acid-base extraction; *p* < 0.05). Based on the physicochemical properties of alkaloids, the acid-base extraction is specifically used to obtain extracts enriched with alkaloids. However, the acid-base extracts displayed a significantly lower yield compared to the other extraction methods.

### 2.3. Thin Layer Chromatography (TLC) Analysis

**Figure 2:**
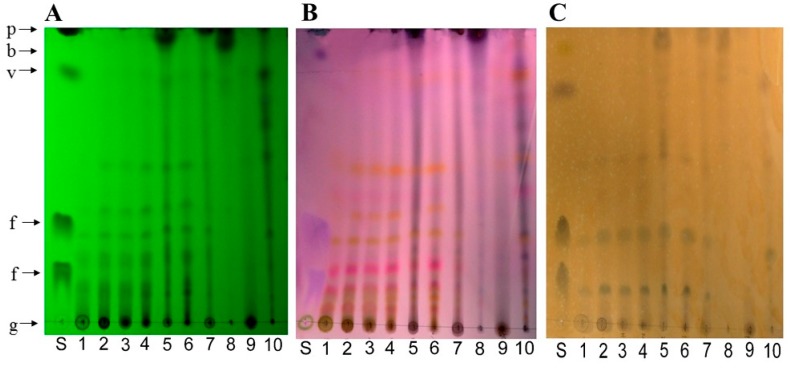
TLC plate demonstrating the phytochemical profile of quaking aspen. TLC was executed using three revelation methods: (**A**) UV light at 254 nm; (**B**) *p*-anisaldehyde reagent; (**C**) iron chloride (FeCl_3_). The different extracts (1–10) represent quaking aspen extracts with different extraction solvents. From left to right: (1) water, (2) methanol, (3) ethanol, (4) acetone, (5) methylene chloride, (6) ethyl acetate, (7) chloroform, (8) hexane, (9) water-ethanol, (10) acid-base extract. (S) corresponds to a standard mix of glucose (g), ferrulic acid (f), vanillin (v), betulin (b) and piperine (p) with their respective localisation on TLC.

Initially, the chemical profile of Quaking Aspen bark extracts was determined using thin layer chromatography coupled with different revelation methods. Compounds of the same family have, in the majority of cases, similar polarities, and thus may have a comparable retention factor (R_f_) value. Also, compounds from the same family can be revealed in the presence of a specific revelation agent. Figure 2 shows the chemical profile of the extracts according to different revelation methods. Overall, polyconjugated compounds were revealed under UV light at 254 nm (Figure 2A). The majority of organic compounds were revealed in different colors using the *p*-anisaldehyde reagent, a universal stain for nucleophile and oxygenated compounds (Figure 2B); phenolic compounds were revealed in orange color using the FeCl_3_ reagent (Figure 2C); and the Dragendorff reagent exposed nitrogenous compounds including alkaloids (data not shown) [22].

The three TLC plates in Figure 2 show that the chemical profile was very similar for extracts (1) water, (2) methanol, (3) ethanol, (4) acetone and (6) ethyl acetate. For instance, similitudes between spots at R_f_ values of ~0.04, 0.10, 0.19, 0.29, 0.38 and 0.52 were apparent. In addition, these extracts had several spots at the bottom of the silica sheet at the same Rf as polyphenol and sugar standards i.e., under an R_f_ value of ~0.83. Conversely, the methylene chloride (5) and hexane (8) extracts displayed spots at the same level as betulin and piperine standards and a few spots at the bottom of the plate. The methylene chloride (5), chloroform (7), hexane (8) and acid-base (10) extracts had similar profiles with trails rather than well-defined spots. The water-ethanol (9) extract contained few compounds with a larger spot present at the bottom the silica sheet. Also, all extracts showed similar phenolic compounds profile except for the extracts with hexane (8) and water-ethanol (9) (Figure 2C), which contained low amounts of phenolic compounds. For instance, the apparent spots in the other extracts had the same R_f_: ~0.12, 0.28 and 0.56. Finally, the use of the Dragendorff reagent did not reveal spots in extracts, but revealed the alkaloid piperine standard, confirming the effectiveness of this revelation method.

### 2.4. Antimicrobial Assays

The antimicrobial activity of quaking aspen bark extracts was tested in vitro using the broth microdilution method to determine the minimal inhibitory concentration (MIC) and the minimal bactericidal/fungicidal concentration (MBC/MFC). Serial dilutions of concentration ranging from 0.01 mg/mL to 4.44 mg/mL were assayed. The results presented in Table 1 show the antimicrobial activity of different extracts against eight pathogenic bacteria and fungi species. The rating of antimicrobial efficiency of extracts was realized according to a proposed classification from Algiannis et al. (2001) based on MIC value as follows: strong inhibitor with MIC < 0.5 mg/mL, moderate inhibitor with MIC between 0.6–1.5 mg/mL and weak inhibitor with MIC > 1.6 mg/mL [23]. The results obtained using the water extract revealed the highest efficiency against all microorganisms compared to other extracts (Table 1). The water extract showed a moderate efficiency against *S. enterica*, *P. aeruginosa* and *A. niger* with an MIC of 0.83 mg/mL and a weak efficiency against *E. coli*, *E. faecalis* and *S. aureus* (MIC of 1.67 mg/mL) as well as *S. cerevisiae* (MIC of 2.22 mg/mL). A bactericidal effect against *S. aureus*, *P. aeruginosa* and *S. cerevirsiae* was also observed according to the MBC/MFC values of 1.67, 4.44 and 4.44 mg/mL, respectively (Table 1). The methanol extract showed a weak antimicrobial activity against *S. aureus*, *A. niger* and *S. cerevisiae* strains with MIC values of 4.44, 1.67 and 2.22 mg/mL, respectively. The methanol extract also had bactericidal activity against *S. cerevisiae* with an MFC value of 4.44 mg/mL (Table 1). Finally, the acid-base extract showed a weak inhibition of *E. coli*, *P. aeruginosa*, *A. niger* and *C. albicans* with MIC values of 4.44, 2.22, 2.22 and 4.44 mg/mL, respectively (Table 1). However, no bactericidal properties were observed with this acid-base extract. None of the other extracts tested demonstrated considerable antimicrobial activity (Table 1).

### 2.5. UPLC-QTOF-MS Characterization

In order to identify compounds present in most effective antimicrobial extracts (namely, those extracted with water and methanol), UPLC-QTOF-MS analyses using positive and negative ionization mode were performed. The masses obtained (showed in Table 2 and Table 3) were compared with exact masses from different libraries using the MZMine 2.0 software [24] to identify potential compounds. A summary of the identified compounds following UPLC-QTOF-MS analysis is presented in Table 2 and Table 3 for the methanol and water extracts, respectively. The exhaustive list for each of the masses obtained is shown in supplementary Tables S1–S4. The percentage area in Table 2 and Table 3 was calculated according to data presented in supplementary tables. In total, 92 different molecules were identified with good confidence, i.e., 39 and 53 molecules in methanol and water extracts, respectively. Molecules were grouped by classes, and other compounds of interest not belonging to the selected families were classified as “others”. In both extracts, polyphenols were most abundant and diversified, followed by glycosylated compounds and phenolic acids. Few terpenoids, alkaloids and sugars were also identified, but these classes were less abundant and diversified. According to the number of compounds identified in Table 2 and Table 3, water was a better solvent to extract a wide variety of compounds compared to methanol.

In each class, compounds were classified according to their percentage area. This value represent the area under the peak of the ionized mass (M ± H) at a specific retention time. It depends largely on the ionization efficiency of the molecule. Thus, at a similar concentration, an easily ionized molecule will appear more abundant then a less ionizable one. However, since similar classes of compounds possess similar chemical feature and ionization potential, we can infer relative abundance based on % area. For example, the polyphenols uvarinol and acacetin in the methanol extract were relatively more abundant compared to other polyphenols with a % area >4%. Considering both extracts, two compounds in particular appeared more abundant: oleamide and 4-prenylresveratrol in water extracts with % areas of 9.61 and 7.26, respectively (Table 3).

**Figure 3:**
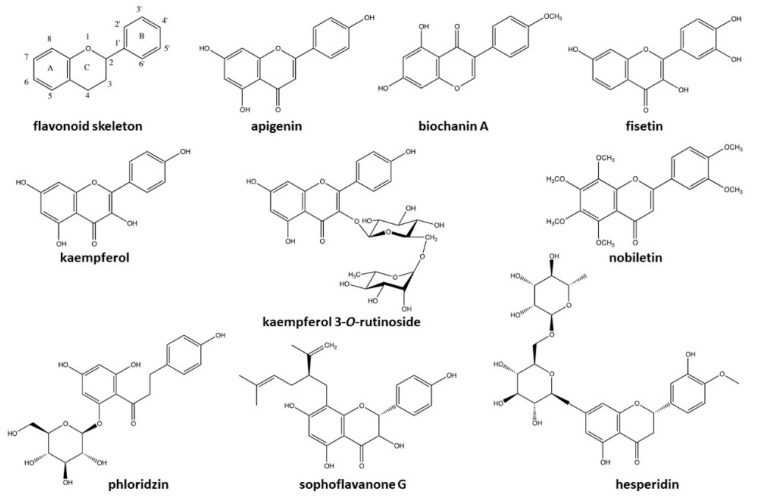
Chemical structure of flavonoids in the quaking aspen extract with potential antimicrobial activity. These flavonoid compounds were proposed by MZMine 2.0 software following UPLC-QTOF-MS analysis and were specifically selected for their antimicrobial potential according to the literature.

Following a literature search on compounds present in both samples, it was possible to target several ones known for their antimicrobial effect. These were grouped and are summarized in Table 4. According to the UPLC-QTOF-MS results (Table 2 and Table 3), all molecules in Table 4 represent 19% and 25.87% of the total % area of the methanol and water extracts, respectively. The grouping of these molecules revealed that the majority of compounds reported for their antimicrobial activity in quaking aspen extracts are polyphenols (eight among the twenty-two identified). In general, the majority of compounds of interest are specific to one extraction solvent, water or methanol, but some were present in both, including catechol, caffeic acid and 4-hydroxybenzaldehyde. Among compounds of interest in the methanol extract, 4-hydroxybenzaldehyde (5.44%), phloridzin (4.76%), and hydroxyanthraquinone (4.05%) were more abundant compared to other compounds based on the % area values (Table 2). For the water extract, kaempferol (5.03%) and its glycosylated form, kaempferol 3-*O*-rutinoside (3.15%), were the most abundant compounds (% area values, Table 3). There was a higher number of different compounds recognized for their antimicrobial property in the water extract than in the methanol extract. Also, nine among the twenty-three molecules targeted were flavonoids (Figure 3), a subgroup of phenolic compounds. In fact, the molecules illustrated in Figure 3, have a similar core structure: apigenin, biochanin A, fisetin, kaempferol, kaempferol 3-*O*-rutinoside, nobiletin, phloridzin, sophoraflavanone G, and hesperidin. The basic structure of flavonoids corresponds to fifteen carbon atom skeleton forming two benzene rings and one bridge formed by three carbons that connect the rings together. This bridge generally forms a pyrone ring as illustrated in Figure 3 (flavonoid skeleton).

## 3. Discussion

### 3.1. Composition and Extraction Yield Evaluation

The aim of this study was to recover, test and identify specialized metabolites from quaking aspen bark in order to obtain antimicrobial extracts. As the final objective is to use raw bark residues from sawmills to produce natural antimicrobials at an industrial scale, it is relevant to characterize this material to further reach an optimal extraction yield. The results obtained from the extraction of each particle size and corresponding ash content revealed that sieving was relevant for eliminating the fractions with a low mass of extractives (Figure 1A,C). In fact, several studies reported that finest particles often contain soil contaminants, such as sand and fine sediments, that yield no extractive organic compounds. This explain the highest ash content observed from the fraction < 3 mm, which represents the inorganic compound proportion in the bark (Figure 1A). Inorganic material contains minerals such as potassium, calcium and sodium [73,74] and these compounds can be harmful to machinery during extraction processes in addition to being undesirable because they do not provide extractive material [75,76]. Thus, better yields of extractives were obtained with bark of particle sizes greater than 3 mm.

Chemical composition tests carried out according to the NREL protocols allowed us to evaluate the composition of the bark material besides the extractives. Results of the average chemical composition of several tree species of the Quebec territory reported by Browne et al. (2009) showed that the bark contained between 16% to 40% cellulose, 38% to 58% hemicellulose, 40% to 50% lignin and 2% to 25% extractive compounds. Results obtained for bark of quaking aspen in the present study were below the average proportions of hemicellulose and lignin, i.e., 13% and 25%, respectively (Figure 1B). Conversely, the residues were composed of 17% extractive materials and this proportion was relatively high compared to the average of tree species in Quebec [77]. In addition, the bark residues had a proportion of cellulose (23%) within the average content of common trees (16–40%). These results demonstrated the relevance to exploit extractive materials from quaking aspen for biorefinery because their abundance is high relative to others tree species from Quebec and also relative to the lignocellulosic compounds of this species.

The extraction with solvents of different dielectric constants, which influence their polarity, allowed extraction of the majority of extractive molecules present in the quaking aspen sawmill residues. Since the extraction yield was significantly higher with polar solvents (Figure 1D), it suggests that abundant specialized metabolites were highly polar compounds, including phenolic compounds, sugars and other glycosylated compounds.

This hypothesis correlated with the chemical profile obtained following the TLC analysis. The three revelation methods (UV, FeCl_3_ and *p*-anisaldehyde) allowed us to determine that a large number of compounds were highly polar by the presence of many spots with a low R_f_ (Figure 2). The FeCl_3_ revelation demonstrated that quaking aspen extracts contain many phenolic compounds (Figure 2C). Finally, revelation with the *p*-anisaldehyde reagent suggests that extracts have compounds similar to sugars based on the presence of spots with the same color and migration as a glucose standard, i.e., green and with an R_f_ value around 0 (Figure 2B). Furthermore, according to Prado and Meireles (2010), green spots with low R_f_ values could be associated with flavonoid compounds.

### 3.2. Antimicrobial Activity

MIC and MBC/MFC results showed that three extracts (water, methanol and acid-base) had antimicrobial properties (Table 1). The water extract followed by the methanol extract yielded a higher efficacy against pathogens. More specifically, the water extract displayed a weak to moderate inhibitory effect on all eight pathogens tested. The larger broad-spectrum antimicrobial activity of this extract could be explained by the diversity of metabolites it contains, as demonstrated by the UPLC-QTOF-MS analysis. Broad-spectrum antimicrobial activity of different plant extracts has been also observed in recent studies [78,79,80]. The results are also consistent with those obtained by [81], who tested the bacteriostatic and bactericidal effects of quaking aspen bud extracts obtain with a methanol extraction against many different pathogens. Quaking aspen extracts had no bacteriostatic effect against Gram-negative bacteria in both studies (*E. coli*, *P. aeruginosa* and *S. enterica*), although Vardar-Ünlü et al. (2008) observed an antimicrobial effect against *E. faecalis* and *C. albicans* while no antimicrobial effect was observed on these pathogens in our study. These differences can be explained by the use of different tree parts that can contain different metabolites at different concentrations. The difference in efficacy against *C. albicans* can also be explained by the difference of the yeast strain used.

Other efforts need to be deployed to maximize efficiency of quaking aspen extracts. In fact, the antimicrobial effect of these extracts was, on average, 800 to 4000 time lower than reference antimicrobial compounds used as active ingredient in drugs and disinfectants. In fact, the MIC range obtained for QAC (reference compound) in this study was 0.651–10.4 μg/mL. Also, recent and older studies reported that the MIC values associated with amphotericin B and cefotaxime, widespread antimicrobials used to treat fungal and bacterial infections, respectively, were between 0.1 and 16 μg/mL against several reference strains [82,83,84,85]. In regards to isolated compounds from plant species that demonstrated strong antimicrobial activity, the gap of MIC values with quaking aspen bark extracts is smaller. For example, MIC values for resveratrol and berberine were between 50 μg/mL and 500 μg/mL [86,87]. Finally, results obtained in this study were in concordance with those obtained for plant extracts in general. For example, Assob et al. (2011) and Khan et al. (2014) evaluated the antimicrobial activity of several plant extracts and the average MIC range was 300–20,000 μg/mL, corresponding to weak or moderate efficiency [79,88]. Isolated compound are generally more effective than extracts. However, an extract with a mix of several compounds can counteract resistance mechanisms through the various mechanisms of action accomplished by the multiple molecules against microorganism targets [89,90,91]. Thus, according to the desired applications, extracts could be relevant in the development of antimicrobial agents.

### 3.3. Chemical Characterization

The UPLC-QTOF-MS analyses enabled the potential identification of the compounds present in water and methanol extracts. These results (Table 2 and Table 3) coupled with TLC revealed the FeCl_3_ reagent (Figure 2C), which showed that the water and methanol extraction allowed the recovery of an important variety of phenolic compounds. Interestingly, several studies reviewed the importance of polyphenols and phenolic acids in extracts responsible for antimicrobial activity [13,48,92,93]. These results substantiated the antimicrobial activity obtained in our study with the families of compounds present in water and methanol extracts.

It was possible to observe through the UPLC-QTOF-MS analyses (Table 2 and Table 3) a wide variety of glycosylated compounds. In this case, these glycosylated compounds were, for the vast majority, phenolic compounds bound to a carbohydrate. Depending on the carbohydrate to which they were bound, some of these compounds had different antimicrobial efficiencies. For example, kaempferol, present in methanol extract (Table 2), is recognized for its antimicrobial activity, but kaempferol-hexoside did not seem to have bioactivity against four pathogens (*S. aureus*, *Paeruginosa*, *E. coli and C. albicans*) according to a study by Fattouch et al. (2007) [34]. On the contrary, kaempferol-3-O-rutinoside is a glycosylated compound which appeared to have antimicrobial efficacy against the same four pathogens in previous studies by Panizzi et al. (2002) and Nenaah (2013) [58,60]. These molecules are interesting because their carbohydrate structure serves as a recognition site for cellular specific targets and generally increases their bioactivity [94,95]. Also, these compounds may be of interest in vivo in the presence of enteric enzymes, which have the ability to hydrolyze these structures and remove the glycosylic bond [96,97].

Some terpenoids and alkaloids were also identified in water and methanol extracts, but these compounds are not as diverse as the compound classes mentioned before. However, these are interesting because some compounds of these families are known to have antimicrobial activity, even at low concentration [16,17,98]. In fact, only four alkaloid compounds were identified in both extracts, and these were not very abundant according to the % area observed (Table 2 and Table 3). Firstly, these results corroborate the poor extraction yield obtained with the acid-base method used to concentrate alkaloids (Figure 1D). These results also corroborate those obtained for TLC analysis that showed a low abundance of alkaloid molecules. The characterization of sugars is relevant since they are undesirable compounds for the purpose of this study. Indeed, the carbohydrates can be used as a substrate by the microorganisms and serve as a source of energy for their proliferation. Knowing their retention time (~1.00 min), it would be possible to remove them from the extracts by fractionation and purification methods and induce carbohydrate starvation with respect to the microorganisms [99].

UPLC-QTOF-MS analyses allowed for the identification of molecules associated with antimicrobial activity according to previous studies (see Ref. in Table 4). It has been suggested that these molecules are responsible of the bacteriostatic and bactericidal effects of quaking aspen extracts. In accordance with the UPLC-QTOF-MS results, molecules shown in Table 4 were particularly abundant in the extract. In fact, these represent 19.00% and 25.87% of the total % area for methanol and water extracts, respectively. The higher efficiency of the water extract can be explain by the higher abundance and diversity of antimicrobial compounds in this extract. Based on these results, it was expected we would find an inhibitory effect against pathogens for both extracts given the abundance of potentially antimicrobial compounds they contain. Antimicrobial properties can be related to specific molecules in Table 4, i.e., 4-hydroxybenzaldehyde in methanol extract and kaempferol in water extract, because they seem to be highly abundant in extracts according to % area results in Table 2 and Table 3.

The grouping of compounds recognized for their antimicrobial effectiveness allowed us to establish a potential relationship between their chemical structure and their activity. These revealed that several molecules among all those present are in the flavonoid class (Figure 3). Thereby, there appears to be a close relationship between antimicrobial activity and the flavonoid structure. This relationship corroborates previous findings by Cushnie and Lamb (2005), Rauha et al. (2000) as well as Sohn et al. (2004), and supports the idea of an important antimicrobial efficiency of these molecules [98,100,101].

It remains difficult to attribute the antimicrobial activity to a specific compound in such a complex compound mixture. Synergistic and antagonistic effects may be present which make analyses sophisticated [89,102]. Also, the study of antimicrobial metabolites from natural plant sources remains laborious due to the variability of the compound concentration between extracts from different raw materials. In fact, metabolite profiles can change according to the quaking aspen species collected, harvest location and/or harvest period, etc. [103,104]. Finally, some compounds remained unidentified due to the detection limit of the UPLC-QTOF-MS apparatus, the library used and the limit of available information. Consequently, it is possible that some compounds with high activity may have been discarded.

## 4. Materials and Methods

### 4.1. Plant Material

The plant material used for this experiment was quaking aspen (*Populus tremuloides*) bark, obtained from sawmill residues of T.L.T Industry in Ste-Monique, Lac-St-Jean, Quebec, Canada, in winter 2016 and winter 2017. The material was dried after reception at room temperature (~20 °C) to preserve it until extraction.

### 4.2. Microbial Strains

The bacterial and fungal strains were obtained from the American Type Culture Collection (ATCC) and from the microbiology laboratory of the University of Québec in Trois-Rivières (UQTR). The microorganisms used were *Escherichia coli* (ATCC 35218), *Salmonella enterica* (ATCC 10708), *Pseudomonas aeruginosa* (ATCC 15442), *Staphylococcus aureus* (ATCC 6538), *Enterococcus faecalis* (ATCC 29212), *Aspergillus niger* (ATCC 10535), *Candida albicans* (from UQTR microbiology laboratory), *Saccharomyces cerevisiae* (from UQTR microbiology laboratory). The bacterial strains were grown on sterilized Mueller Hinton agar and incubated at 37 °C for 24 h, while the fungal strains were grown on Sabouraud dextrose agar and incubated at 37 °C for 72 h before use.

### 4.3. Determination of Bark Composition and Extractive Yield per Fraction Size

To determine the general composition and extractive yield of quaking aspen bark residues per fraction size, sieving was first performed. The following sieve mesh sizes were used: 3 mm, 7 mm, 45 mm, to obtain four different fractions: (a) <3 mm, (b) 3–7 mm, (c) 7–45 mm, (d) >45 mm. The bark was then ground in a Wiley Mill crusher at 0.425 mm for further extraction and analysis.

For the general composition, procedures from the National renewable energy laboratory (NREL) were carried out. Specifically, the NREL/TP-510-42620 and NREL/TP-510-42622 protocols were followed on fraction sizes <3 mm and >3 mm to dry and weigh the total biomass, and to calculate ash abundance, respectively. A water and ethanol (85.75% EtOH, 13.7% MeOH, 0.85% ethyl acetate; (Fisher scientific, Ottawa, ON, Canada) extraction using an accelerated solvent extraction apparatus (ASE) (Dionex ASE-350, ThermoFisher scientific, Ottawa, ON, Canada) was carried out for the determination of extractible abundance on fractions >3 mm following protocol NREL/TP-510-42619. Finally, determination of the structural compound abundance was realized on fractions > 3 mm following the NREL/TP-510-42618 protocol with some modifications. Cellulose and hemicellulose were fractioned by acid hydrolysis, and quantification of monomeric sugars was realized by ionic chromatography (Dionex ICS-5000, ThermoFisher scientific, Ottawa, ON, Canada) with a Dionex CarboPac SA-10 column, while lignin was quantified by UV-VIS spectrometry (DR6000, Hach, London, ON, Canada) [105].

To determine which particle size fraction of raw bark residues contained the highest amount of extractives, three aliquots of 100 g of each fraction (3–7 mm, 7–45 mm, >45 mm) was used to perform extraction using an ASE apparatus with three different solvents: (1) distilled water, (2) water-ethanol and (3) ethanol. The yield was determined by measuring the extractive material mass of each fraction.

### 4.4. Extractive Yield According to Molecular Polarity

Bark residues with a fraction size greater than 3 mm were used to prepare ten aliquots of 10 g of dried bark powder (0.425 mm). Each was extracted with different solvents having distinct polarities: (1) distilled water, (2) methanol (99.9%; HPLC-grade; Fisher scientific), (3) denaturised ethanol, (4) acetone (99.8%; Fisher scientific; certified ACS), (5) methylene chloride (>99%; Acros organics), (6) ethyl acetate (99.6%; Acros organics; certified ACS), (7) chloroform (99.9%. Acros organics), (8) hexane (99.9%; Fisher chemical; HPLC-grade), (9) water-ethanol, and (10) by specific solvents for acid-base extraction method. For samples 3, 4, 5, 6, 7 and 8, the extraction was done with a Soxhlet system for 7 h over six cycles of extraction. For samples 1, 2 and 9, the extraction was performed using ASE. The water extraction was performed for 70 min (6 cycles of 10 min), at 100 °C and at a pressure of 1500 psi, and the ethanol extraction was performed for 45 min (6 cycles of 5 min) at 120 °C and 1500 psi. For the water-ethanol extraction, the matrix was first extracted with water and then extracted with denaturised ethanol with the same parameters as mentioned before according to each extraction solvent. Finally, the acid-base extraction method (liquid-liquid extraction) was performed as described by Yubin (et al. (2014)) [106].

The extraction yield was determined by measuring the dried extractive material mass for each solvent. After that, each extract was suspended in two aliquots, one in DMSO for antimicrobial analysis (99.8%; Fisher scientific; certified ACS) and the other in the respective solvent of extraction for chemistry analysis, both at a concentration of 10 mg/mL. These stock solutions were stored in the fridge at 4 °C until further analysis. All extractions were performed in triplicate.

### 4.5. Thin Layer Chromatography Method (TLC)

For a rapid visualization of the extract’s chemical profile, TLC analysis was carried out. For this purpose, a TLC plate (silica gel 60 F_254_ in aluminium, Sigma, Saint-Louis, MO, USA) with a dimension of 14 × 20 cm was used. A solution with standards belonging to different families of compounds such as piperine, betulin, vaniline, ferrulic acid and glucose, all at 1000 ppm, was prepared. Development was realized in a presaturated solvent chamber with CHCl_3_-MeOH-EtOH (90:9:1). For visualisation purpose, the TLC plate was first observed under UV light at 254 nm and was also sprayed with *p*-anisaldehyde reagent, following by heating on a hot plate at 105 °C for 5 min. This solution was prepared by following the mixture proposed in Prado and Meireles (2010) [107]. A similar procedure of TLC plate development was further performed but was sprayed with different reagents: FeCl_3_ (for phenolic compounds revelation) using a modified method based on a protocol from SiliCycle Inc. (2017) by mixing 1.6 g of FeCl_3_, 50 mL of water and 50 mL of methanol, and secondly with Dragendorff reagent (for alkaloid revelation) following the mixture proposed by Jia and Tian (2009) [22,108].

### 4.6. Antimicrobial Activity Test by Broth Microdilution Method

To determine the minimum inhibitory concentration (MIC) of extracts, a broth microdilution method described in documents M07-A7 and M38-A2, adapted from the Clinical and Laboratory standard institute (CLSI), were used [109,110].

First, microbial colonies were collected from an agar plate and suspended in a sterilized solution of physiological water (9 g of NaCl in 1 L of distilled water) to obtain a final cell concentration of 0.5 Mc Farland, corresponding to 1.5 × 10^8^ CFU/mL for bacteria, 1.5 × 10^6^ CFU/mL for yeasts and 1.5 × 10^4^ CFU/mL for molds. In a 96-well plate, 100 µL of tested extracts in DMSO solution (10 mg/mL) was deposited in wells of the first column. Then, 50 µL of Mueller Hinton broth was added to each well, and a serial dilution was realized by transferring 50 µL of the first well into the second of the same row and so on to obtain a serial dilution of the extracts ranging between 4.44 to 0.01 mg/mL. Subsequently, 50 µL of the microbial inoculum (0.5 Mc Farland, [110]) was then added to the wells. In each microplate test, a positive control of quaternary ammonium compound (QAC) BTC 6358 solution (1.19 g/L), obtained from the sanitation products company Sani Marc (Victoriaville, QC, Canada), was used. The plate was incubated at 37 °C for 3 h for the bacterial strains and 6 h for fungal strains. After incubation, a solution of INT (2-p-iodophenyl-3-p-nitrophenyl-5-phenyl tetrazolium chloride (Sigma, Saint-Louis, MO, USA) at 2.85 mg/mL was added. This tetrazolium salt, reduced to a formazan red dye by the coenzyme NADH of living cells, served as a cell viability indicator. The plate was incubated a second time to reduce the tetrazolium salt (1 h for the bacterial strains/16 h for the fungal strains). The MIC (minimum inhibitory concentration) was determined in the first well of the serial dilution where no color appeared, which indicates that there is inhibition of microbial proliferation. The non-toxic effect of DMSO at 33% (maximal concentration used during the microdilution test) was evaluated, and the absence of an inhibition effect confirmed the non-toxic effect of the matrix used to dilute the extracts.

To optimise the protocol, screening was performed initially with each bark extract to rapidly determine which ones provided the inhibition of microbial proliferation at maximum concentration (4.44 mg/mL), and only effective extracts were kept for the microdilution analysis. For determination of the minimum bactericidal/fungicidal concentration (MBC/MFC), 100 µL of each well containing extracts with a positive effect on proliferation during the microdilution broth test was plated on the agar surface with the respective growth medium (Mueller Hinton for bacteria and Sabouraud dextrose for fungi). The Petri dishes were incubated at 37 °C for 24 h for the bacterial strains and 72 h for the fungal strains. The MBC and MFC were determined by the lowest concentration that allowed no visible colony on the agar Petri dish, i.e., more than a 99.9% reduction in colonies.

### 4.7. UPLC-QTOF-MS Method

UPLC-QTOF-MS analyses were carried out externally by the industrial research center of Québec (CRIQ). Briefly, analysis was performed with a Waters Acquity ultra-performance LC system (Waters, Milford, MA, USA). This apparatus is equipped with a binary pump system model (Waters). The separation was achieved using an Ethylene Bridged Hybrid (BEH) C18 column (100 mm × 2.1 mm id. 1.7 mm particle size), also from Waters. The UPLC system was coupled with a Waters QTOF micro mass spectrometer (Waters, Milford, MA, USA) equipped with a z-spray electrospray interface for the MS analysis. The mobile phase used was two eluents: A = 0.2% acetic acid, B = acetonitrile (99.9% HPLC grade). Elution parameters were as follows: solvent flow rate 0.2 mL/min; injection volume 10 µL; proportions of eluent B: 2% (0–1 min); 2–100% (1–30 min); isocratic 100% (30–33 min); 100–2% (33–33.5 min), isocratic 2% (33–40 min). The MS analysis was performed in both positive and negative modes, and the data were acquired from 100 to 1250 *m*/*z* without collision. The conditions of the ionization source were established as follows: source temperature 120 °C; cone gas flow rate 50 L/h; desolvation gas flow rate 350 L/h; desolvation gas temperature 200 °C; cone voltage 30 V in negative mode and 70 V in positive mode; capillary voltage 1150 V in negative mode and 1800 V in positive mode. Ultra high purity nitrogen (99%) was used as a nebulizing gas. Data acquisition was carried out with the Masslynx 4.1 software (Waters, Milford, MA, USA). Mass extraction, deconvolution, isotopes and the library search were performed using MZMine 2.0 according to Pluskal et al. (2010) [24]. The molecule identification was determined by the concordance of the *m*/*z* values with the theoretical values using different libraries databases: Chemspider, Kegg Compounds Database, LipidMaps Database and the CRIQ database.

### 4.8. Statistical Analysis

One-way analysis of variance (ANOVA) was performed on data with a 5% level of probability (*p* < 0.05) using JMP software [24] followed by pairwise mean comparison Tukey’s test was conducted where differences were detected.

## 5. Conclusions

The aim of this study was to determine the antimicrobial potential of quaking aspen bark residues through the extraction of bioactive metabolites. We found that specialized metabolite extractions are relevant to isolate antimicrobial molecules from bark residues. The extraction yield results as well as MIC and MBC/MFC results identified two promising solvents: water and methanol. The physicochemical analysis of these two extracts identified hundreds of metabolites of interest, among which some had an antimicrobial effect in accordance with previous studies. Analysis of the chemical structure of these compounds revealed possible correlations between flavonoid structure and antimicrobial effect.

Overall, our study supports the relevance of being interested in quaking aspen bark residues to valorize its biomass through these bioactive metabolites. Until now, this species was undervalued because no previous exhaustive study linked the chemical composition of its bark to its antimicrobial activity. However, this study allowed us to confirm the antimicrobial efficacy by the presence of antimicrobial compounds in quaking aspen. This species is also interesting because optimal extraction conditions are consistent with antimicrobial efficacy results. Water, which is the most efficient solvent, is the solvent suitable for extraction since it is non-polluting, inexpensive and the most accessible.

## Figures and Tables

**Table 1:** Antimicrobial activity of quaking aspen bark extracts against different strains of microorganisms using the broth microdilution method. The ATCC numbers are *E. coli* (ATCC 35218), *S. enterica* (ATCC 10708), *P. aeruginosa* (ATCC 15442), *S. aureus* (ATCC 6538), *E. faecalis* (ATCC 29212), *A. niger* (ATCC 10535). *C. albicans and S. cerevisiae are strains from the microbiology Laboratory at the Université du Québec à Trois-Rivières*.

Extract	*E. coli*		*S. enterica*		*P. aeruginosa*		*S. aureus*		*E. faecalis*		*A. niger*		*C. albicans*		*S. cerevisiae*	
	MIC ^a^	MBC ^b^	MIC	MBC	MIC	MBC	MIC	MBC	MIC	MBC	MIC	MFC	MIC	MFC	MIC	MFC
Water	1.67	− ^c^	0.83	−	0.83	4.44	1.67	1.67	1.67	−	0.83	−	1.67	−	2.22	4.44
Methanol	−	−	−	−	−	−	4.44	−	−	−	1.67	−	−	−	2.22	4.44
Ethanol	−	−	−	−	−	−	−	−	−	−	−	−	−	−	−	−
Acetone	−	−	−	−	−	−	−	−	−	−	−	−	−	−	−	−
Methylene chloride	−	−	−	−	−	−	−	−	−	−	−	−	−	−	−	−
Ethyl acetate	−	−	−	−	4.44	−	−	−	−	−	−	−	−	−	−	−
chloroform	−	−	−	−	−	−	−	−	−	−	−	−	−	−	−	−
Hexane	−	−	1.67	−	−	−	−	−	−	−	−	−	−	−	−	−
Water-ethanol	−	−	−	−	−	−	−	−	−	−	−	−	−	−	−	−
Acid-base	4.44	−	−	−	2.22	−	−	−	−	−	2.22	−	4.44	−	−	−
QAC ^d^	2.60 ^e^	5.21 ^e^	0.651 ^e^	0.651 ^e^	10.4 ^e^	10.4 ^e^	5.21 ^e^	5.21 ^e^	2.60 ^e^	2.60 ^e^	10.4 ^e^	10.4 ^e^	5.21 ^e^	5.21 ^e^	2.60 ^e^	2.60 ^e^

^a^ MIC, minimum inhibitory concentration. Values given as mg. mL^–1^ for extracts and as mg. L^–1^ for QAC; ^b^ MBC, minimum bactericidal concentration/MFC, minimum fungicidal concentration. Values given as mg. mL^−1^ for extracts and as mg. L^–1^ for QAC; ^c^ Not active at maximum concentration (4.44 mg. mL^–1^); ^d^ QAC; quaternary ammonium compound as a positive control; ^e^ Concentration expressed in mg. L^–1^.

**Table 2:** Summary of the characterization of quaking aspen compounds of interest identified from methanol extracts following UPLC-QTOF-MS analysis.

Compound Class	Proposed Compound ^a^	% Area ^b^	RT ^c^ (min)	Exact Mass (*m*/*z*)	
				[M + H]^+ d^	[M − H]^− d^
Polyphenols	Uvarinol	4.44	13.01		573.1666
	Acacetin	4.37	15.73		285.0872
	Cyanidin	2.22	7.71	288.017	
	Chamuvaritin	1.8	9.70		451.1318
	Hesperidin	1.76	13.32		301.084
	Dimethylquercetin	1.57	6.06		329.1043
	Catechol	1.22	5.99		109.0582
	Acetylglycitin	0.95	9.09		487.1998
Phenolic acids	Diferuloyquinic acid	4.19	12.41		543.1303
	Coumaric acid	1.73	7.9		163.0654
	Hydroxybenzoic acid	1.13	9.96		137.0445
	Coumaroylquinic acid	1.05	5.17		337.1691
	Caffeic acid	0.78	1.31		179.0711
Terpenoids	Arbusculin A	1.38	8.15	251.16	
	Confertifolin	1.36	6.57	235.1504	
	Palustradiene	0.76	8.93	273.2144	
Sugars	Galloyl glucose	4.82	5.29		331.1157
	D-Ribofuranose	1.53	6.11	150.869	
Glycosylated compounds	Phloridzin	4.76	10.93		435.1344
	Apigenin-glucoside	4.22	10.42		431.14
	Grandidentatin	3.89	9.94		423.1767
	Coumaric acid glucoside	3.45	6.21		325.0998
	Naringenin-glucoside	1.36	9.34		433.1198
	Luteolin-hexoside	0.81	11.49		447.1463
	Kaempferol-hexoside	0.75	10.72		461.1678
	Kaempferol-hexoside	0.71	9.67		447.1667
Alkaloids	Dihydrozeatin	3.75	10.44	222.1829	
	isoquinoline-1.5-diol	3.14	5.34	161.7838	
	(−)-Hygroline	1.54	7.15	144.1	
Others	Butonate	5.47	13.95	326.9853	
	4-Hydroxybenzaldehyde	5.44	9.83	122.8692	
	1,2,4-Trimethylbenzene	4.19	15.79	121.5811	
	Hydroxyanthraquinone	4.05	10.95	225.053	
	Gluconic acid	2.06	13.98	195.1299	
	Adipic acid	1.65	5.22	146.8463	
	Nobiletin	0.98	1.45		401.1542
	Malic acid	0.97	8.67	133.4128	
	Thiodiacetic acid	0.89	13.69	150.9699	
	1.4-Naphthoquinone	0.83	1.52	159.0468	

^a^ Proposed compounds were based on a match of exact mass results with base data in the MZMine 2.0 library using the UPLC-QTOF-MS method. Some compounds were discarded from the list because they did not abide by the following selection criteria: (1) % area above 0.5%; (2) compounds whose exact structure was identified by the library (compounds with a crude formula only were discarded); (3) recognized compounds in literature, mainly for their biological activity; ^b^ % area was calculated for each ionization mode analysis; ^c^ RT; Retention time; ^d^ [M + H]^+^: exact mass from positive ionization mode analysis. [M – H]^−^: exact mass from negative ionization mode analysis.

**Table 3:** Summary of the characterization of quaking aspen compounds of interest identified from water extracts following UPLC-QTOF-MS analysis.

Compound Class	Proposed Compound ^a^	% Area ^b^	RT ^c^ (min)	Exact Mass (*m*/*z*)	
				[M + H]^+ d^	[M − H]^− d^
Polyphenols	4-prenylresveratrol	7.26	14.4		295.1533
	Kaempferol	5.03	15.6	287.0954	
	Isorhamnetin	4.39	10.63		315.1832
	3-methoxyapigenin	3.49	15.9	301.0837	
	Cirsimaritin	3.13	11.46		313.1609
	Pinobanksin	3.11	12.2	273.0878	
	Epirosmanol	2.58	5.08		345.1393
	Medioresinol	2.19	7.34		387.1865
	Kaempferide	2.12	14.1		299.187
	Kaempferol	1.98	15.56		285.0911
	Catechol	1.63	5.65		109.0384
	Sophoraflavanone G	1.45	10.13		423.1942
	5-tricosenylresorcinol	1.39	10.25		431.1579
	Kaempferide	1.21	15.93		299.0782
	Fisetin	1.08	12.4	287.0953	
	Apigenin	0.92	13.6	271.1084	
	Biochanin A	0.92	15.7	285.0903	
	Glepidotin	0.68	15.23		297.175
	Apigenin	0.51	12.2	271.0766	
Phenolic acids	4-hydroxybenzoic acid	2.09	9.15		137.0339
	3-hydroxybenzoic acid	1.77	5.58		137.0339
	Caffeic acid	0.94	1.18		179.0467
	4-hydroxyphenylacetic acid	0.83	4.35		151.0183
	Hydroxycaffeic acid	0.67	1.21		195.0708
	Vanilic acid	0.59	6.37		167.0509
Terpenoids	Trilobolide	1.13	11.51		521.2712
	Phytuberin	0.87	15.31	293.1385	
Sugars	D-Rhamnose	2.64	1	165.0511	
	Chitobiose	1.01	9.8	425.1993	
	D-Glucose	0.65	1	181.0349	
Glycosylated compounds	Kaempferol 3-*O*-rutinoside	3.15	11.08		593.2896
	Salicin	2.37	11.4	287.1035	
	Syringin	1.93	11.9	373.1422	
	Myricetin 3-*O*-arabinoside	1.74	5.58		449.1708
	Arbutin	1.65	12.2		271.0804
	Jaceidin 4′-*O*-glucuronide	1.47	9.67		535.2496
	Ferulic acid 4-*O*-glucoside	1.33	9.93		355.1625
	Fisetin 8-C-glucoside	1.19	9.1	449.1325	
	Quercetin-3-*O*-glucuronide	0.8	9.3		477.251
	Gallic acid 4-*O*-glucoside	0.8	14.4		331.1295
	Quercetin-3-*O*-glucuronide	0.68	10.16		477.251
Alkaloids	Berberine	0.5	11.9	337.1196	
Others	Oleamide	9.61	26.6	282.2869	
	Octadecyl	3.38	23.8	254.2584	
	1-Heptadecanamine	2.89	26	256.276	
	4-Hydroxybenzaldehyde	1.47	9.6	123.0455	
	4-Hydroxybenzaldehyde	1.11	6.85		121.0385
	Coumarin	1.03	7.6	147.0472	
	4-nitrophenol	0.96	34.9	139.9936	
	Benzoin	0.89	5.1	213.1013	
	Simazine	0.76	1	202.0671	
	Diosgenin	0.75	7.2	415.276	
	1.4-Naphthoquinone	0.55	3.14		157.0664

^a^ Proposed compounds were based on a match of exact mass results with base data in the MZMine 2.0 library using the UPLC-QTOF-MS method. Some compounds were discarded from the list because they did not abide by the following selection criteria: (1) % area above 0.5%; (2) compounds whose exact structure was identified by the library (compounds with a crude formula only were discarded); (3) recognized compounds in literature, mainly for their biological activity; ^b^ % area was calculated for each ionization mode analysis; ^c^ RT; Retention time; ^d^ [M + H]^+^: exact mass from positive ionization mode analysis. [M − H]^−^: exact mass from negative ionization mode analysis.

**Table 4:** Examples of compounds with known antimicrobial properties identified in Quaking Aspen bark extracts.

Compound Class	Compound Name ^a^	Presence ^b^		Ref.
		Water Extract	Methanol Extract	
Polyphenols	Hesperidin	−	+	[25,26]
	Catechol	+	+	[27,28]
	Medioresinol	++	−	[29,30]
	Sophoraflavanone G	+	−	[31,32,33]
	Kaempherol	+++	−	[34,35,36]
	Fisetin	+	−	[37,38]
	Apigenin	+	−	[39,40,41]
	Biochanin A	+	−	[36,42]
Phenolic acids	Coumaric acid	−	+	[43,44,45]
	Caffeic acid	+	+	[46,47,48,49,50]
	Vanillic acid	+	−	[46,50,51,52]
	4-hydroxybenzoic acid	++	−	[46,53]
	3-hydroxybenzoic acid	+	−	[51,52]
Terpenoids	Confertifolin			[54,55]
Glycosylated compound	Phloridzin	−	+++	[49,56,57]
	Kaempferol 3-*O*-rutinoside	++	-	[58,59,60]
Alkaloids	Berberine	+	−	[61,62,63]
	Nobiletin	−	+	[26,64]
Others	Malic acid	−	+	[65,66,67]
	Hydroxyanthraquinone	−	+++	[67,68,69]
	4-hydroxybenzaldehyde	+	+++	[70,71]
	Coumarin	+	-	[71,72]

^a^ Presented compounds were selected according to their antimicrobial activity demonstrated in recent studies, and this activity has been demonstrated against at least two pathogens; ^b^ Relative abundance according to the % area value reported in Table 2 and Table 3. +++: >4%; ++: 2–4%; +: 0.5–2%; −: 0% (absent).

## Supplementary Materials

The following are available online at http://www.mdpi.com/1420-3049/23/7/1739/s1, Supplementary tables: Tables S1–S4.
