# Supplementary material for: Phytochemical Screening of Quaking Aspen (Populus tremuloides) Extracts by UPLC-QTOF-MS and Evaluation of their Antimicrobial Activity
Source: Molecules. 2018 Jul 16;23(7):1739. doi: 10.3390/molecules23071739 (PMC6099928; doi:10.3390/molecules23071739)
Supplement: Supplementary file 1 [file molecules-23-01739-s001.pdf]

molecules

Supplementary data : St-Pierre, A. et al. 2018

Table S1. Full data of metabolites (M-H) from quaking aspen methanol extract obtained following UPLC-QTOF-MS analysis with negative ionization mode.

Exact mass (M-H)	Rt	Potential identity	Area under curve
271,069	12,41	Naringenin, Phloretin, Butein ou Arbutin	2852,9
287,066	9,75	Eriodictylol isomer ou Phlorin	2191,7
331,1158	5,29	Galloyl glucose	2115,7
435,1344	10,93	Phloridzin	2090,5
573,1666	13,01	$C_{36}H_{30}O_7$-Uvarinol	1951
285,0872	15,73	Acacetin	1918,8
431,14	10,42	Apigenin-glucoside	1849,7
543,1304	12,41	Diferuloylquinic acid	1839
423,1767	9,94	$C_{21}H_{28}O_9$-Grandidentatin	1707,1
469,1442	8,64	Valoneic acid dilactone	1533,2
421,1602	9,21	$C_{21}H_{26}O_9$	1519,5
325,0998	6,21	Coumaric acid glucoside	1513,7
345,1339	5,29	$C_{16}H_{26}O_8$	1308,5
393,1098	13,92	$C_{19}H_{22}O_9$	953,9
521,1724	7,68	$C_{28}H_{26}O_{10}$	895,7
575,1824	12,2	Dimethylquercetin	853,8
451,1318	9,7	Chamuvaritin	790,5
301,084	13,32	Hesperidin	773,1
163,0654	7,9	Coumaric acid	758,8
319,0613	7,06	3,4-DHPEA-EDA	717,3
571,2123	5,3	$C_{30}H_{36}O_{11}$	695,1
329,1043	6,06	Dimethylquercetin	687
377,1151	14,71	3,4DHPEA-EA	645,7
499,149	16,24	$C_{26}H_{28}O_{10}$	618,2
433,1198	9,34	Naringenin-glucoside	598,8
393,1194	12,73	$C_{19}H_{22}O_9$	570,3
323,1502	5,2	$C_{14}H_{28}O_8$	564,6
109,0582	5,99	Catechol	537,4
137,0445	9,96	Hydroxybenzoic acid	497,2
561,2155	9,94	$C_{29}H_{38}O_{11}$	487,7
321,1371	4,73	Gallic acid gallate	465,7
337,1691	5,17	Coumaroylquinic acid	462,3
335,157	4,69	$C_{15}H_{28}O_8$	434,8
401,1542	1,45	Nobiletin	428,4

Table S1 (continued). Full data of metabolites (M-H) from quaking aspen methanol extract obtained following UPLC-QTOF-MS analysis with negative ionization mode.

Exact mass (M-H)	Rt	Potential identity	Area under curve
487,1998	9,09	Acetylglycitin	416,9
439,1776	8,79	$C_{22}H_{32}O_9$	413
575,136	9,73	Methyllathosterol ferulate ou Procyanindin Dimer	383,6
447,1463	11,49	Luteolin-hexoside	356,4
303,067	9,4	Taxifolin	351,7
424,2017	10,02	$C_{22}H_{33}O_8$	347
179,0711	1,31	Caffeic acid	342,9
483,1639	18,33	$C_{26}H_{28}O_9$	331,3
461,1678	10,72	Kaempferol-hexoside	330,2
863,2891	10,38	Procyanidin Trimers	329,2
439,127	8,79	$C_{24}H_{24}O_8$	318,8
447,1667	9,67	Kaempferol-hexoside	311,7
329,2622	13,63	$C_{15}H_{38}O_7$	309,3
424,1917	9,6	$C_{22}H_{33}O_8$	242,2
359,1403	1,34	Lariciresinol	236,7
863,3459	10,38	Procyanidin Trimers	229,7
447,1769	6,8	Kaempferol-hexoside	219,4
575,194	11,03	Methylenecholestanol ferulate ou Procyanindin Dimer	204,3
449,1829	10,89	Eriodictyol-hexoside	203
571,2007	15,75	$C_{30}H_{36}O_{11}$	171

Table S2. Full data of metabolites (M+H) from quaking aspen methanol extract obtained following UPLC-QTOF-MS analysis with positive ionization mode.

Exact mass (M-H)	Rt	Potential identity	Area under curve
249,6912	8,63		8364,6
326,9853	13,05	Butonate	7292,8
122,8692	9,83	4-Hydroxybenzaldehyde	7257,8
152,2319	5,36	$C_8H_{39}O$	6517,6
121,5811	15,79	1,2,4-Trimethylbenzene	5590,1
225,0530	10,95	Hydroxyanthraquinone	5405,9
148,1674	6,27	$C_{10}H_{27}$	5179,5
216,4697	9,95		5134,7
222,1829	10,44	Dihydrozeatin	5001,9
215,0282	9,23	2-Deoxy-D-ribose 1-phosphate	4515,5
161,7838	5,34	isoquinoline-1,5-diol	4190,2
288,0170	7,71	Cyanidin (pigment coloré)	2954,8
227,9560	8,86	H_3O_{14}	2746,1
195,1299	13,98	Gluconic acid	2745,3
327,7507	13,10		2626,8
216,4768	10,33	$C_6H_{47}O_6$	2540,5
215,0353	9,52	2-Deoxy-D-ribose 5-phosphate	2357,9
146,8463	5,22	Adipic acid	2194,1
216,4483	8,64	$C_{14}H_{47}$	2097,6
144,1000	7,15	(-)-Hygroline	2053,8
150,8690	6,11	D-Ribofuranose	2043,3
200,8027	1,53		2011,6
233,7107	9,68		1957,6
251,1600	8,15	Arbusculin A	1835,5
292,4727	13,04		1826,0
235,1504	6,57	Confertifolin	1817,6
250,4251	8,68	$C_6H_{49}O_8$	1814,9
271,7062	16,28		1776,4
259,9468	8,66	$C_9H_7O_9$	1732,8
226,5058	7,38		1710,7
156,3452	5,22	C_9H_{47}	1616,7
145,4903	4,74		1504,2
217,1958	9,97	12-Hydroxydodecanoic acid	1325,9
133,4128	8,67	Malic acid	1289,1
215,7519	9,24		1273,1
233,7070	4,90		1268,7

Table S2 (continued). Full data of metabolites (M+H) from quaking aspen methanol extract obtained following UPLC-QTOF-MS analysis with positive ionization mode.

Exact mass (M-H)	Rt	Potential identity	Area under curve
150,9699	13,69	Thiodiacetic acid	1182,3
152,9186	5,31	$C_{10}O_2$	1147,0
150,8749	6,97	Sodium iodide	1110,1
159,0468	1,52	1,4-Naphthoquinone	1101,6
277,6432	4,99		1071,1
267,3015	5,01	$C_7H_{38}O_9$	1055,8
273,2144	8,93	Palustradiene	1016,8
148,8508	6,26		987,8
132,0816	8,66	Benzoyl cyanide	978,6
238,0878	10,26	$C_7H_9O_9$	856,3
268,7578	6,59		814,0
217,1958	9,66	12-Hydroxydodecanoic acid	790,9
141,4313	4,58	$C_5H_{48}O_2$	788,1
162,4732	5,32		774,3
340,6401	12,65		719,1
228,6865	8,87		687,1
262,8858	8,34	$C_3H_2O_{14}$	619,6
328,5082	13,01		609,7
183,8970	1,44		577,5
293,2339	13,04	Grevillol	535,8
141,4313	4,20	C_8H_{44}	407,8
130,7846	4,86		395,0
242,3964	8,65	$C_8H_{49}O_6$	363,5
217,1744	8,67	12-Hydroxydodecanoic acid	357,3
201,5089	1,52		316,0
262,8780	8,04	$C_3H_2O_{14}$	271,7
126,1111	36,43	gamma-Coniceine	236,5

Table S3. Full data of metabolites (M-H) from quaking aspen water extract obtained following UPLC-QTOF-MS analysis with negative ionization mode.

Exact mass (M-H)	Rt	Potential identity	Area under curve
295,1533	14,40	4-Prenylresveratrol	5410,3
315,1832	10,63	Isorhamnetin	3273,7
459,2164	7,24	5-Pentacosylresorcinol	2653,7
329,2584	13,55	$C_{21}H_{30}O_3$	2593,8
593,2896	11,08	Kaempferol 3-O-rutinoside	2346,3
313,1609	11,46	Cirsimaritin	2328,5
537,2649	9,21	$C_{32}H_{42}O_7$	1923,4
345,1393	5,09	Epirosmanol	1919,8
311,1451	13,05	Caffeoyl tartaric acid	1731,1
387,1865	7,34	Medioresinol	1631,6
299,1870	14,10	Kaempferide	1578,4
137,0339	9,15	4-Hydroxybenzoic acid	1558,7
285,0911	15,56	Kaempferol	1473,0
423,1893	9,79	2,2-Dimethyl-3-(4-methoxyphenyl)-4-ethyl-7-hydroxy-2H-1-benzopyran-8-methanol diacetate	1425,3
327,1651	7,33	p-Coumaroyl tyrosine	1332,1
153,0332	4,37	Gallic aldehyde	1322,9
137,0339	5,58	3-Hydroxybenzoic acid	1319,9
449,1708	11,92	Myricetin 3-O-arabinoside	1299,6
271,0804	12,20	Arbutin	1228,9
109,0384	5,65	Catechol	1214,6
591,3069	14,43	Sitosterol ferulate	1133,8
163,0541	7,60	4-Coumarate	1103,0
535,2496	9,67	Jaceidin 4'-O-glucuronide	1095,6
423,1942	10,13	Sophoraflavanone G	1082,8
431,1579	10,25	5-Tricosenylresorcinol	1035,9
337,1743	5,01	3-p-Coumaroylquinic acid	1025,3
355,1625	9,93	Ferulic acid 4-O-glucoside	990,7
299,0783	15,93	Kaempferide	899,3
615,2812	9,18	Octyl 3,6-di-O-(alpha-D-mannopyranosyl)-beta-D-mannopyranoside	895,1
521,2712	11,51	Trilobolide	846,0
121,0385	6,85	4-Hydroxybenzaldehyde	828,8
669,3165	8,89	Spinacetin 3-O-glucosyl-(1->6)-glucoside	824,8
285,0666	12,40	Fisetin	804,2
537,2144	6,27	6-O-methyl-N-deacetylisoipecoside	799,7
191,0339	1,31	p-Coumaric acid ethyl ester	745,9

Table S3 (continued). Full data of metabolites (M-H) from quaking aspen water extract obtained following UPLC-QTOF-MS analysis with negative ionization mode.

Exact mass (M-H)	Rt	Potential identity	Area under curve
179,0467	1,18	Caffeic acid	700,6
609,2938	8,84	Quercetin 3-O-xylosyl-glucuronide	676,1
187,1118	9,57	Homo-L-arginine	673,6
487,2146	9,10	6''-O-Acetylglycitin	657,1
475,2254	9,65	Ellagic acid acetyl-arabinoside	648,9
293,1385	15,31	Phytuberin	645,5
507,2165	9,96	Gibberellin 2-O-beta-D-glucoside	644,7
133,0229	1,34	(S)-Malate	641,0
471,2184	8,56	Deoxylimonate	625,0
151,0184	4,35	4-Hydroxyphenylacetic acid	618,7
117,0299	2,05	Succinate	604,2
331,1295	14,40	Gallic acid 4-O-glucoside	598,9
477,2510	9,30	Quercetin 3-O-glucuronide	594,1
487,2093	9,41	6''-O-Acetylglycitin	580,7
653,3156	11,05	adipic acid; 2,2-bis(hydroxymethyl)butyl benzoate; isophthalic acid; pentane-1,5-diol	539,2
297,1750	15,23	Glepidotin C	509,0
477,1982	10,16	Quercetin 3-O-glucuronide	506,0
195,0708	1,21	Hydroxycaffeic acid	502,2
287,0781	9,52	Phlorin	499,1
335,1533	4,51	2-Butoxy-2-oxoethyl butyl phthalate	477,1
165,1039	8,76	Methoxyphenylacetic acid	475,7
549,2317	9,42	Quercetin 3-O-(6	469,3
325,1308	9,12	Feruloyl tartaric acid	455,9
167,0509	6,37	Vanillic acid	440,1
465,1720	9,53	Dihydromyricetin 3-O-rhamnoside	438,7
135,0546	7,60	p-Anisaldehyde	437,8
165,0542	1,21	Methoxyphenylacetic acid	426,2
205,1783	22,67	Acetyl eugenol	417,4
571,2360	5,09	(1aR,1bS,4aR,7aS,7bS,8R,9R,9aS)-4a,7b-Dihydroxy-3-(hydroxymethyl)-1,1,6,8-tetramethyl-[…][a]	411,7
157,0664	3,14	1,4-Naphthoquinone	407,5
343,0677	0,96	5-O-Galloylquinic acid	397,5
421,2471	34,87	Lovastatin acid	396,9
405,1702	6,22	Piceatannol 3-O-glucoside	392,7
627,3199	11,46	Gambogic acid	384,4
171,1150	11,62	Decanoic acid	382,3
325,1308	10,47	Feruloyl tartaric acid	340,8

Table S3 (continued). Full data of metabolites (M-H) from quaking aspen water extract obtained following UPLC-QTOF-MS analysis with negative ionization mode.

Exact mass (M-H)	Rt	Potential identity	Area under curve
283,1760	7,96	Geraldone	338,6
157,1391	17,85	1,4-Naphtoquinone	319,7
461,2493	11,54	5-Pentacosylresorcinol	288,8
201,0444	0,94	Bergaptol	284,9
421,1380	5,30	Picrasin F	276,3
493,2357	8,48	Microlenin	257,3
309,1358	13,08	Cinnamoyl glucose	236,9
491,2299	8,87	Isorhamnetin 3-O-glucuronide	214,4

[a](1aR,1bS,4aR,7aS,7bS,8R,9R,9aS)-4a,7b-Dihydroxy-3-(hydroxymethyl)-1,1,6,8-tetramethyl-5-oxo-1,1a,1b,4,4a,5,7a,7b,8,9-decahydro-9aH-cyclopropa[3,4]benzo[1,2-e]azulene-9,9a-diyl dibenzoate

Table S4. Full data of metabolites (M+H) from quaking aspen water extract obtained following UPLC-QTOF-MS analysis with positive ionization mode.

Exact mass (M+H)	Rt	Potential identity	Area under curve
287,0954	9,5	Fisetin	13755,2
282,2869	26,6	Oleamide	12785,6
287,0954	15,6	Kaempferol	6690,1
301,0838	15,9	3-Methoxyapigenin	4642,4
254,2584	23,8	Octadecyl	4501,2
563,5732	26,6	Oleic acid, eicosyl ester	4161,9
273,0878	12,2	Pinobanksin	4142,8
256,2760	26,0	1-Heptadecanamine	3839,0
165,0511	1,0	D-Rhamnose	3514,9
287,1035	11,4	Salicin	3157,5
228,2430	23,0	1-Dodecylguanidine	3050,1
781,2897	10,8	3,3'-(Methylenedi-4,1-phenylene)bis(2,4,5-triphenyl-2,4-cyclopentadien-1-one)	2566,1
373,1422	11,9	Syringin	2565,2
107,0503	5,1	Aromatic aldehyde	2488,9
184,0484	1,0	4-Pyridoxate	2402,3
142,0390	1,0	Carbamoyl phosphate	2216,7
564,3776	8,2	N,N'-Didodecyl-6-(trichloromethyl)-1,3,5-triazine-2,4-diamine	2203,9
520,3510	7,9	$C_{32}H_{45}N_3O_3$	2182,7
123,0455	9,6	4-Hydroxybenzaldehyde	1959,8
608,4004	8,5	9-sec-Butyl-6-(1-ethyl-1H-indol-3-yl)-3-(6-oxooctyl)octahydro-[…][a]	1928,2
476,3257	7,5	4-Amino-3-{[3-amino-6-(aminomethyl)-3,4-dihydro-2H-pyran-2-yl]oxy}-6-[...][b]	1808,5
449,1325	9,1	Fisetin 8-C-glucoside	1585,8
652,4316	8,7	1-Deoxy-1-[dodecanoyl(nonyl)amino]-4-O-hexopyranosylhexitol	1536,5
279,1578	5,0	2-Ethylhexyl phthalate	1451,6
408,1826	10,8	$C_{25}H_{29}NO_4$	1438,7
503,3298	7,9	$C_{33}H_{42}O_4$	1391,4
147,0472	7,6	Coumarin	1365,7
425,1993	9,8	Chitobiose	1346,2
459,2998	7,5	11,17-Dihydroxy-3,20-dioxopregna-1,4-dien-21-yl 3,3-dimethylbutanoate	1340,6
547,3581	8,2	Tri-2-octanyl 1,2,4-benzenetricarboxylate	1328,7
373,1422	9,8	Syringin	1311,8
139,9936	34,9	4-Nitrophenol	1276,6
193,0568	8,2	Citrate	1270,2

Table S4 (continued). Full data of metabolites (M+H) from quaking aspen water extract obtained following UPLC-QTOF-MS analysis with positive ionization mode.

Exact mass (M-H)	Rt	Potential identity	Area under curve
573,2514	5,1	adipic acid; 2-ethylhexanoic acid; 2-ethyl-2-(hydroxymethyl)propane-1,3-diol; isobenzofuran-1,3-dione	1252,2
546,2267	13,8	3-Glycoloyl-3,5,12-trihydroxy-10-methoxy-6,11-dioxo-1,2,3,4,5a,6,11,11a-octahydro-1-tetracenyl 3-amino-2,3,6-trideoxyhexopyranoside	1244,3
271,1084	13,6	Apigenin	1220,3
285,0903	15,7	Biochanin A	1218,2
213,1013	5,1	Benzoin	1182,6
140,0050	1,0	4-Nitrophenol	1169,0
268,2797	25,2	3-(Tetradecyloxy)propanenitrile	1146,0
432,3024	7,2	2-[4-(7-Methoxy-2,2-dimethyl-3-phenyl-3,4-dihydro-2H-chromen-4-yl)phenoxy]-N,N-dimethylethanamine	1095,8
375,1638	7,2	Secologanate	1048,3
107,0503	12,0	D-Glycerate	1017,1
202,0671	1,0	Simazine	1012,4
415,2709	7,2	Diosgenin	999,7
339,1214	10,1	(-)-Glyceollin I	963,6
253,0986	10,3	1-Hydroxy-2,3-dimethyl-9,10-anthraquinone	939,6
591,3834	8,4	1,4:5,9-Dianhydro-1-[9-(2-carboxy-3-hydroxy-4-methylphenyl)-6-hydroxy-[…]	923,7
696,4679	8,9	3-(2-Naphthyl)-N-palmitoyl-L-alanyl-L-alpha-glutamyl-L-leucine	903,2
181,0349	1,0	D-Glucose	867,0
161,0354	1,0	2-Oxoadipate	836,4
249,0927	12,0	1-Hydroxy-6-methoxypyrene	813,5
105,0374	10,8	Hydroxypyruvate	811,0
815,3554	9,8	$C_{42}H_{54}O_{16}$	736,6
423,1863	9,3	$C_{25}H_{26}O_{6}$	727,8
271,0766	12,2	Apigenin	684,2
393,1235	14,3	1,5-Diphenoxy-anthraquinone	680,9
337,1196	11,9	Berberine	668,3
149,0311	22,5	trans-Cinnamate	619,8
181,0349	34,9	D-Hexose	612,4
124,0296	1,0	Nitrobenzene	602,1
119,0215	1,0	Succinate	600,7
245,0922	9,5	Gentisein	561,2
284,0619	1,0	2-tert-Butyl-4,6-dinitrophenyl carbamate	560,0
167,0524	1,0	D-Xylonate	554,1
798,3342	10,8	$C_{41}H_{51}NO_{15}$	541,8

Table S4 (continued). Full data of metabolites (M+H) from quaking aspen water extract obtained following UPLC-QTOF-MS analysis with positive ionization mode.

Exact mass (M-H)	Rt	Potential identity	Area under curve
373,1422	10,8	Syringin	531,1
415,1526	10,3	10-Hydroxy-5-(3,4,5-trimethoxyphenyl)-5,8,8a,9-tetrahydrofuro[3',4':6,7]naphtho[2,3-d][1,3]dioxol-6(5aH)-one	498,9

[a]9-sec-Butyl-6-(1-ethyl-1H-indol-3-yl)-3-(6-oxooctyl)octahydro-2H-pyrido[1,2-a][1,4,7,10]tetraazacyclododecine-1,4,7,10(3H,12H)-tetrone

[b]4-Amino-3-{[3-amino-6-(aminomethyl)-3,4-dihydro-2H-pyran-2-yl]oxy}-6-(ethylamino)-2-hydroxycyclohexyl 3-deoxy-4-C-methyl-3-(methylamino)pentopyranoside

[c]1,4:5,9-Dianhydro-1-[9-(2-carboxy-3-hydroxy-4-methylphenyl)-6-hydroxy-5,7-dimethyl-4-oxononan-3-yl]-2,3,6,7-tetradeoxy-4-ethyl-8-C-ethyl-2,9-dimethylnonitol
